# DETERMINANTS OF INFORMAL CARE TRAJECTORIES IN ENGLAND

**DOI:** 10.1093/geroni/igad104.1622

**Published:** 2023-12-21

**Authors:** Giorgio Di Gessa, Christian Deindl

**Affiliations:** University College London, London, England, United Kingdom; TU Dortmund University, Dortmund, Nordrhein-Westfalen, Germany

## Abstract

In the context of an ageing population, combined with long-standing challenges in the delivery of formal social care for older people, unpaid caregivers play a key role in promoting the quality of life of older people and their extended families and ensuring that needs for care and support are met. Although many studies have provided snapshots of informal care provision, few so far have examined longitudinal patterns of informal care provision among older people. This paper aims to describe caregiving trajectories in later life and to examine how socioeconomic, demographic, and health characteristics of older adults relate to these patterns (including needs and enabling factors). Using six waves of the English Longitudinal Study of Ageing, we conduct latent trajectory analysis to cluster people’s diverse trajectories into a finite number of groups. The intensity of informal care provision is also considered when identifying longitudinal trajectories. Preliminary results show five distinct trajectories of informal care provision (including those who never provide informal care, those who provide sporadic informal care, those with increasing and decreasing commitment to care provision, and those who care throughout). Gender, age, health, income, living arrangements, and family compositions all relate to long-term trajectories of informal care, with younger married women in good health more likely to provide informal care throughout. Support should be provided to this group of caregivers, given that engagement in long-term caregiving might be detrimental to mental health.

